# Supplementary material for: Time-resolved observation of band-gap shrinking and electron-lattice thermalization within X-ray excited gallium arsenide
Source: Sci Rep. 2015 Dec 11;5:18068. doi: 10.1038/srep18068 (PMC4676029; doi:10.1038/srep18068)
Supplement: Supplementary Information [file srep18068-s1.pdf]

Time-resolved observation of band-gap shrinking and electron-lattice thermalization within X-ray excited gallium arsenide

– Supplemental Material –

Beata Ziaja[1,2*], Nikita Medvedev[1], Victor Tkachenko[1],

Theophilos Maltezopoulos[3], and Wilfried Wurth[1,3,4]

[1] *Center for Free-Electron Laser Science CFEL,*
Deutsches Elektronen-Synchrotron DESY,
Notkestrasse 85, 22607 Hamburg, Germany

[2] *Institute of Nuclear Physics, Polish Academy of Sciences,*
Radzikowskiego 152, 31-342 Kraków, Poland

[3] *Department of Physics and Center for Free-Electron Laser Science CFEL,*
University of Hamburg, Luruper Chaussee 149, 22761 Hamburg, Germany

[4] *DESY Photon Science, Deutsches Elektronen-Synchrotron DESY,*
Notkestrasse 85, 22607 Hamburg, Germany

(Dated: April 29, 2015)

Calculation Method

In the paper we use Drude model extended for the interband transitions to calculate the transient reflectivity change from the dielectric function ϵ which is the function of optical coefficients, n and k. In what follows SI units are used. The dielectric function is parametrized as in Ref. [1]:

$$\epsilon \equiv (n + i\,k)^2 = \epsilon_{core} - \sum_{j=e,h} \frac{\omega_{p,j}^2}{\omega_\gamma^2} \frac{(\omega_\gamma \tau_j)^2}{(\omega_\gamma \tau_j)^2 + 1} + \sum_{j=e,h} i\, \frac{\omega_{p,j}^2}{\omega_\gamma^2} \frac{\omega_\gamma \tau_j}{(\omega_\gamma \tau_j)^2 + 1}, \quad (1)$$

* Corresponding authors: B. Ziaja (ziaja@mail.desy.de) and N. Medvedev (nikita.medvedev@desy.de).

where ϵ_{core} describes all contributions to the dielectric function beyond the free-carrier absorption. Here, $\epsilon_{core} = (n_{core} + i\, k_{interband})^2$, where $k_{interband} = \alpha\, \lambda_\gamma / 4\pi$ (λ_γ is a photon wavelength) describes the contribution from the transition between valence and conduction bands, using the interband absorption coefficient for a direct interband transition α parametrized as in Ref. [1] (Eq. (5.31) therein). It reads:

$$k_{interband} = \frac{e^2\,(2\,m_r)^{3/2}}{m^2\, n_{core}\, (\hbar\omega_\gamma)^2\, \hbar\, \epsilon_0}\, |\, \langle v|p|c\rangle\, |^2\, \sqrt{\hbar\omega_\gamma - E_{gap}}\, [f(E_v) - f(E_c)]\, |_{E_c - E_v = \hbar\omega_\gamma}\,. \quad (2)$$

Coefficient m_r denotes the reduced mass for carriers within conduction band and within valence band, i.e., $1/m_r = 1/m_c + 1/m_v$, where $m_c = m_e$ and $m_v = m_h$ are effective electron and hole masses. Matrix element $\langle v|p|c\rangle$ couples states with the same electron wave vector in the valence and conduction bands. We parametrize it, using the measured absorption coefficients for GaAs from Ref. [2] (Fig. 3 therein). The factor $\sqrt{\hbar\omega - E_{gap}}$ originates from the joint density of states evaluated at the critical point M_0 [1]. As a result the absorption coefficient scales with the photon energy $E_{phot} = \hbar\omega_\gamma$ as $\alpha \sim \sqrt{E_{phot} - E_{gap}}/E_{phot}$. Factor $[f(E_v) - f(E_c)]\, |_{E_c - E_v = \hbar\omega_\gamma}$ is a state population weight: it contains Fermi functions representing transient occupation of states within valence and conduction bands with the condition imposed that $E_c - E_v = \hbar\omega_\gamma$. Assuming simple parabolic bands which is accurate enough for states close to band's top/bottom, one obtains expressions for E_c and E_v as a function of band gap E_{gap} and photon energy $\hbar\omega_\gamma$:

$$\begin{aligned} E_v &= -E_{gap}/2 - \hbar^2\, k^2/(2\, m_v) \\ E_c &= +E_{gap}/2 + \hbar^2\, k^2/(2\, m_c), \end{aligned} \quad (3)$$

where $k^2 = \frac{2\, m_r}{\hbar^2}\, (\hbar\omega_\gamma - E_{gap})$.

To sum up, if Eq. (1) is used for the calculation of the transient dielectric function, the following parameters have to be provided: photon frequency ω_γ, transient plasma frequencies for electrons and holes $\omega_{p,e(h)}$, electron and hole collision times $\tau_{e(h)}$, real part of the refraction index of unperturbed system n_{core}, and the calculated value of $k_{interband}$ which, in particular, depends on the photon energy $E_{phot} = \hbar\, \omega_\gamma$ and the actual band gap E_{gap}.

As described in the paper, the evolution of the irradiated system proceeds in two stages. During the excitation stage that begins with X-ray laser irradiation of GaAs, electrons

and holes are produced. This occurs within a few hundred femtoseconds since the exposure start. These timescales are too short for recombination and thermal exchange with lattice to significantly contribute [3, 4]. Therefore, we can neglect these processes during the excitation stage. Also, the density of electrons and holes n_{e-h} increases almost linearly with time [5, 6]. We can therefore describe it with an approximate linear rate equation:

$$d\,n_{e-h}(t)/dt \;=\; \gamma_{e-h}(t), \tag{4}$$

where the coefficient γ_{e-h} depends on the pulse fluence and has been adjusted so as to obtain the maximal value of n_{e-h} corresponding to the number of absorbed X-ray photons divided by the average electron-hole pair creation energy $\langle E_{e-h}\rangle$ which is 4.2 eV for GaAs [7]. E.g., for the case of hard X-ray irradiation with 800 eV photons in [8] it yields: $n_{e-h} = 2.47\cdot 10^{20}, 4.95\cdot 10^{20}, 9.90\cdot 10^{20}$ cm^{-3} for pulse fluences $F = 10, 20, 40$ mJ/cm^2, respectively.

Evolution of the electron kinetic temperature is strongly non-equilibrium during the first tens of femtoseconds after the exposure [5]. The temperature strongly decreases and then stabilizes. However, only a very low number of electrons (maximally of the order of a few permilles of the initial valence electron density) is excited. Also, at such short timescales there is no significant thermal exchange with the lattice yet. We therefore do not need to follow in detail the electron temperature during the excitation stage. Wherever it is needed, we estimate it by assuming its slow linear growth with time from the room temperature (300 K) to a temperature T_e^{init} (at the minimum of $\Delta R/R$ curve). We initially fix T_e^{init} from the relation quoted in the text: $\langle T_e^{init}\rangle \approx 2/3\,(\langle E_{e-h}\rangle - E_{gap})$, assuming negligible kinetic energy of heavy holes. Later we iteratively fit it to the data.

Let us mention that as the lattice temperature does not change much during the first $100-200$ fs after the FEL irradiation, i.e., during electron thermalization, the band-gap width does not change either during this time.

Knowing the real part of the refraction index from unperturbed system n_{core} and the transient carrier densities, we can calculate $k_{interband}$ from Eq. (2). As the carrier densities n_{e-h} determine the plasma frequency of the material $\omega_{p(e,h)} \sim \sqrt{n_{e-h}}$, the plasma frequencies $\omega_{p,e(h)}$ for electrons and holes can then be calculated, and with them the transient complex refraction index from Eq. (1). With this, transient reflectivity can be directly obtained (see, e.g., [9]). The average electron collision time τ_e is then fitted iteratively until the reflectivity curve at the end of the first evolution stage matches the minimum of the experimental $\Delta R/R$

curve. The average hole collision time τ_h can then be estimated from the electron one, using mass scaling relation for electron and hole collision frequencies [10]. The product of collision time and photon frequency is $\omega \cdot \tau_e \sim 1$ for 800 nm and $\omega \cdot \tau_e \sim 2$ for 400 nm.

As shown in Ref. [6, 11] the minimum of the $\Delta R/R$ curve corresponds to the maximal electron-hole density in the sample. Reaching the maximal electron-hole density within the system concludes the excitation stage of the sample evolution. At later times the relaxation stage begins, and proceeds on picosecond timescales. Free carrier distribution can then be assumed to be thermal. The recombination of the electron-hole pairs begins to contribute as well as the electron-lattice thermalization. The rate equation for electron-hole distribution takes the recombination into account:

$$d\,n_{e-h}(t)/dt = -\gamma_{rec} \cdot n_{e-h}(t), \tag{5}$$

where γ_{rec} is the recombination rate including both radiative and nonradiative recombination coefficients for GaAs taken from [3]. Equilibration of electrons and lattice is described by the set of temperature equations for lattice temperature T_{latt} and electron temperature T_{el}:

$$d\,T_{latt}(t)/dt \;=\; +G_{latt}[T_{el}(t) - T_{latt}(t)] \tag{6}$$

$$d\,T_{el}(t)/dt \;=\; -G_{el}[T_{el}(t) - T_{latt}(t)], \tag{7}$$

where we assumed spatially uniform electron and lattice temperatures with no temperature gradients and neglected any source terms, as the system only relaxes. As in GaAs holes are very heavy, the electrons will predominantly contribute to the lattice heating. Therefore, we neglect the contribution of holes in the temperature equations. With coefficients G_{latt} and G_{el} the electron-lattice thermalization time can be defined as $\tau_{el-latt} = 1/(G_{latt} + G_{el})$. The coefficients G_{latt} and G_{el} are related to heat capacities of the system through the relations: $G_{latt\,(el)} = G/C_{latt\,(el)}$, where C_{el} is the heat capacity of the free-electron gas taken from Ref. [10] and C_{latt} is the heat capacity of the lattice taken from Ref. [7]. The coefficient G is a temperature-dependent electron-phonon coupling factor. Using previous relations, it can be expressed as: $G = C_{el} \cdot C_{latt}/[(C_{el} + C_{latt})\,\tau_{el-latt}]$. Band-gap shrinkage (entering Eqs. (2), (3) and consequently, Eq. (1)) with increasing lattice temperature is described with the phenomenological relation from [7]. The initial conditions for the equations at the end of the excitation stage are: $T_{latt} = 300$ K and $T_{el} = T_e^{init}$.

Similarly as we did during the first evolution stage, we calculate transient reflectivity and adjust iteratively $\tau_{el-latt}$ and the free electron temperature T_e^{init} at the minimum of $\Delta R/R$

curve, so as to obtain the best fit to the experimental curve. However, we keep collision times fixed. In Table 1 (from the main text) we listed the final parameters obtained. The values lay within the range reported in Ref. [12]. Note also the decrease of the thermalization time with the electron temperature [12].

We assign the dependence of T_e^{init} on the fluence to the strong non-isothermality of the conduction and valence band carriers that maintains up to a few hundred femtoseconds since the exposure to the FEL pulse and even after the carriers within each band already thermalized. This non-equilibrium process cannot be accurately described with our approximate model and would require dedicated non-equilibrium ab-initio studies.

References

---

[1] M. S. Dresselhaus. Solid state physics II: optical properties of solids. *http://web.mit.edu/course/6/6.732/www/6.732-pt2.pdf.*

[2] M. D. Sturge. *Phys. Rev.*, 127:768, 1962.

[3] B. E. A. Saleh and M. C. Teich. Fundamentals of Photonics. *J.Wiley and Sons, ISBN: 0-471-83965-5*, 1991.

[4] N. Medvedev, Z. Li, and B. Ziaja. in press. *Phys. Rev. B*, 2015.

[5] B. Ziaja, D. van der Spoel, A. Szöke, and J. Hajdu. *Phys. Rev. B*, 64:214104, 2001.

[6] N. Medvedev et al. *Contr. Plasma Phys.*, 53:347, 2013.

[7] Database on Semiconductors. http://www.ioffe.rssi.ru/SVA/NSM/Semicond/.

[8] O. Krupin et al. *Opt. Exp.*, 20:11396, 2012.

[9] P. Yeh. Optical waves in layered media. *J. Wiley and Sons, ISBN: 0-471-73192-7*, 2005.

[10] N.W. Ashcroft and N.D. Mermin. Solid state physics. *Harcourt, Inc.*, 1976.

[11] M. Harmand et al. *Nat. Phot.*, 7:215, 2013.

[12] N. Del Fatti et al. *Phys. Rev. B*, 59:4576, 1999.
